# Perioperative Immunonutrition in Gastrointestinal Oncology: A Comprehensive Umbrella Review and Meta-Analysis on Behalf of TROGSS—The Robotic Global Surgical Society

**DOI:** 10.3390/nu17142304

**Published:** 2025-07-13

**Authors:** Aman Goyal, Christian Adrian Macias, Maria Paula Corzo, Vanessa Pamela Salolin Vargas, Mathew Mendoza, Jesús Enrique Guarecuco Castillo, Andrea Garcia, Kathia Dayana Morfin-Meza, Clotilde Fuentes-Orozco, Alejandro González-Ojeda, Luis Osvaldo Suárez-Carreón, Elena Ruiz-Úcar, Yogesh Vashist, Adolfo Pérez Bonet, Adel Abou-Mrad, Rodolfo J. Oviedo, Luigi Marano

**Affiliations:** 1 Department of General Surgery, Mahatma Gandhi Medical College and Research Institute, Puducherry 607402, India; doc.aman.goyal@gmail.com; 2 Department of Surgery, Adesh Institute of Medical Sciences and Research, Bathinda 151001, India; 3 School of Medicine, Universidad Catolica de Santiago de Guayaquil, Guayaquil 090615, Ecuador; christianmaciasmd@gmail.com; 4 Department of Health and Sciences, Hillsborough Community College, Tampa, FL 33614, USA; 5 Center for Space Emerging Technologies, Lima 15046, Peru; 6 Department of Surgery, Universidad de Los Andes, Bogota 111711, Colombia; mp.corzo10@uniandes.edu.co; 7 Facultad de Medicina, Universidad Westhill, Mexico City 05610, Mexico; vanesalolin@gmail.com; 8 Department of Surgery, University of Houston Tilman J. Fertitta Family College of Medicine, Houston, TX 77001, USA; mmendo31@cougarnet.uh.edu; 9 Larkin Community Hospital, South Miami, FL 33431, USA; guarecucomd@gmail.com; 10 Unidsad de Investigación Biomédica 02, Hospital de Especialidades del Centro Médico Nacional de Occidente, Guadalajara 44349, Mexico; andreagarcianu@gmail.com (A.G.); kathiamorfin@gmail.com (K.D.M.-M.); clotilde.fuentes@gmail.com (C.F.-O.); 11 Facultad de Medicina, Universidad de Colima, Colima 28040, Mexico; andreagar7007@gmail.com; 12 Department of Bariatric Surgery, UMAE Hospital de Especialidades del Centro Medico Nacional de Occidente, Guadalajara 44349, Mexico; md.suarezcarreon@gmail.com; 13 Department of Surgery, Universidad de Guadalajara, Guadalajara 44340, Mexico; 14 Fuenlabrada Universitary Hospital, Rey Juan Carlos University, 28943 Madrid, Spain; eruizucar@gmail.com; 15 Department of Surgery, Organ Transplant Center for Excellence, Center for Liver Diseases and Oncology, King Faisal Specialist Hospital and Research Center, Riyadh 12271, Saudi Arabia; y.vashist@kfu.edu.sa; 16 Department of Surgery, Clinica Gloria Patricia Pinzón Clinic, Florencia 180001, Colombia; perezadolfo.21md@gmail.com; 17 Department of Surgery, Centre Hospitalier Universitaire d’Orléans, 45000 Orléans, France; adel.abou-mrad@orange.fr; 18 Department of Surgery, Nacogdoches Medical Center, Nacogdoches, TX 75962, USA; 19 Department of Surgery, Sam Houston State University College of Osteopathic Medicine, Conroe, TX 77301, USA; 20 Department of Medicine, Academy of Applied Medical and Social Sciences-AMiSNS: Akademia Medycznych i Spolecznych Nauk Stosowanych, 52-300 Elbląg, Poland; 21 Department of General Surgery and Surgical Oncology, “Saint Wojciech” Hospital, “Nicolaus Copernicus” Health Center, 80-000 Gdańsk, Poland; 22 Department of Surgery, Dnipro State Medical University, 49044 Dnipro, Ukraine; 23 Department of Medicine, Surgery, and Neurosciences, University of Siena, 53100 Siena, Italy

**Keywords:** perioperative immunonutrition, gastrointestinal cancer surgery, postoperative complications, surgical outcomes, systematic review and meta-analysis

## Abstract

Introduction: Gastrointestinal (GI) cancers are associated with high morbidity and mortality. Surgical resection, the primary treatment, often induces immunosuppression and increases the risk of postoperative complications. Perioperative immunonutrition (IMN), comprising formulations enriched with omega-3 fatty acids, arginine, nucleotides, and antioxidants, has emerged as a potential strategy to improve surgical outcomes by reducing complications, enhancing immune function, and promoting recovery. Methods: A systematic search of PubMed, Scopus, and the Cochrane Library was conducted on 28 October 2024 in accordance with PRISMA guidelines. Systematic reviews and meta-analyses evaluating perioperative IMN versus standard care in adult patients undergoing GI cancer surgery were included in the search. The outcomes assessed included infectious and non-infectious complications, wound healing, hospital stay, and nutritional status. The study quality was evaluated using AMSTAR 2, and the meta-analysis was conducted using random-effects models to calculate the pooled effect sizes (risk ratios [RRs], odds ratios [ORs], mean differences [MDs]) with 95% confidence intervals (CIs). Results: Sixteen systematic reviews and meta-analyses, including a total of 41,072 patients, were included. IMN significantly reduced infectious complications (RR: 0.62, 95% CI: 0.55–0.70; *I*^2^ = 63.0%), including urinary tract infections (RR: 0.74, 95% CI: 0.61–0.89; *I*^2^ = 0.0%) and wound infections (OR: 0.64, 95% CI: 0.55–0.73; *I*^2^ = 34.4%). Anastomotic leak rates were notably lower (RR: 0.68, 95% CI: 0.62–0.75; *I*^2^ = 8.2%). While no significant reduction in pneumonia risk was observed, non-infectious complications decreased significantly (RR: 0.83, 95% CI: 0.75–0.92; *I*^2^ = 30.6%). IMN also reduced the length of hospital stay by an average of 1.92 days (MD: −1.92, 95% CI: −2.36 to −1.48; *I*^2^ = 73.5%). Conclusions: IMN provides significant benefits in GI cancer surgery, reducing complications and improving recovery. However, variability in protocols and populations highlight the need for standardization and further high-quality trials to optimize its application and to validate its efficacy in enhancing surgical care.

## 1. Introduction

Gastrointestinal (GI) cancers are among the leading causes of cancer-related morbidity and mortality worldwide, posing significant challenges to healthcare systems. Surgical resection remains a cornerstone of curative treatment; however, the physiological stress of surgery, compounded by the often compromised immune status of patients with GI malignancies, increases the risk of postoperative complications and impairs recovery. In this context, perioperative immunonutrition (IMN) has garnered attention as a promising adjunctive strategy to optimize surgical outcomes [1].

Immunonutrition involves the administration of specialized nutritional formulas enriched with specific nutrients, such as omega-3 fatty acids, arginine, nucleotides, and antioxidants. These components are believed to modulate immune responses, to attenuate inflammation, and to support tissue repair, thereby enhancing recovery in surgical patients [2]. Emerging evidence suggests that IMN can significantly reduce postoperative complications, particularly infectious complications, and improve overall clinical outcomes in high-risk populations, including those undergoing GI cancer surgery. For instance, Matsui et al. demonstrated that perioperative IMN reduced infectious complications by approximately 30% compared to standard nutritional therapy, highlighting its potential in improving surgical care [3].

The benefits of IMN extend beyond infectious complications. In the patients with head and neck cancers, perioperative IMN has been associated with improved wound healing and reduced postoperative infections, as reported by Riso et al. [4]. Similarly, in gastric cancer patients, preoperative IMN was linked to a significant reduction in postoperative infections, as demonstrated in a study by Yu et al. [5]. These findings underscore the potential of IMN to enhance immune resilience and to facilitate recovery during the perioperative period.

The mechanisms underlying these benefits appear to be multifaceted. Omega-3 fatty acids, for example, possess potent anti-inflammatory properties that mitigate the production of pro-inflammatory cytokines, thereby reducing systemic inflammation and the risk of complications [6]. Arginine, a conditionally essential amino acid, has been shown to enhance T-cell functions, promoting an effective immune response, while nucleotides play a crucial role in cell proliferation and tissue repair, accelerating recovery [7]. These nutrient-specific effects collectively contribute to the observed improvements in clinical outcomes.

The patients with GI and head and neck cancers often present with severe nutritional deficits due to the metabolic demands of their malignancies, further exacerbated by surgical stress. Studies have consistently demonstrated that perioperative IMN in these populations not only reduces postoperative infections but enhances wound healing, reduces the length of hospital stay, and facilitates a faster return to normal functions [8,9]. These benefits translate into reduced healthcare resource utilization, including shorter hospitalizations and decreased costs associated with complications and rehabilitation [9].

In addition to improving immune function and wound healing, IMN has been shown to enhance gut barrier integrity, a critical factor in the prevention of systemic inflammation and infection during surgery. Increased intestinal permeability is a known contributor to postoperative complications; by reducing intestinal permeability, IMN mitigates this risk and facilitates better recovery outcomes [10]. Furthermore, its cost-effectiveness makes it an attractive intervention in resource-limited settings, where minimizing complications and optimizing recovery are paramount [11].

Given these promising findings, this study aims to synthesize and critically appraise the current evidence on perioperative IMN in GI cancer surgery. Through an umbrella review of systematic reviews, we evaluate the impact of IMN on key clinical outcomes, including postoperative complications, length of hospital stay, wound healing, and infectious complications. Our goal is to contribute to the understanding of IMN as a valuable adjunct in surgical oncology, providing evidence-based insights for clinicians and researchers seeking to improve outcomes in this complex patient population.

## 2. Materials and Methods

### 2.1. Search Strategy

A comprehensive systematic search was conducted across PubMed, Scopus, and the Cochrane Library on 28 October 2024 to identify systematic reviews and meta-analyses evaluating perioperative IMN in GI cancer, in accordance with the Preferred Reporting Items for Systematic Reviews and Meta-analyses (PRISMA) and the guidelines by Smith et al. [12]. The search strategy employed a combination of keywords and Medical Subject Headings (MeSH) terms, including “immunonutrition”, “gastrointestinal cancer”, and “surgery”. To ensure comprehensive coverage, additional manual searches of the key journals and the reference lists of the identified studies were performed. Grey literature, such as dissertations and conference abstracts, was excluded to maintain a focus on peer-reviewed evidence. Duplicate records were identified and removed using Rayyan software (v1.1.0), streamlining the screening process.

### 2.2. Eligibility Criteria

The studies were selected based on predefined eligibility criteria using the PICO framework. The population included adult patients undergoing surgery for GI cancer, while the studies involving pediatric populations or animals were excluded. The intervention of interest was perioperative IMN strategies, compared with standard perioperative care or no IMN intervention. The clinical outcomes assessed included postoperative complications (both infectious and noninfectious), hospital length of stay, wound healing, and improvements in nutritional status. The eligible studies included systematic reviews or meta-analyses published in peer-reviewed journals in English. Studies were excluded if they were abstracts, editorials, narrative reviews, or preprints.

### 2.3. Study Selection

The study selection process involved an initial screening of the titles and abstracts by two independent reviewers using Rayyan software. Full texts of the potentially eligible studies were retrieved and reviewed in detail. Discrepancies were resolved through consensus or consultation with a third reviewer. This rigorous two-step process ensured the inclusion of high-quality studies that were directly relevant to the research questions.

### 2.4. Data Extraction

Data extraction was conducted independently by two reviewers using a standardized data collection form to ensure consistency and accuracy. The extracted data included study identifiers (author names, title, and publication year), population characteristics (sample size, demographics, and clinical details), and details of perioperative IMN interventions. The clinical outcomes of interest—postoperative infectious complications, postoperative noninfectious complications, length of hospital stay, wound healing, and improvements in nutritional status were systematically documented. All data were cross-checked for accuracy, and disagreements were resolved by consensus.

### 2.5. Assessment of Methodological Quality

The methodological quality of the included systematic reviews and meta-analyses was rigorously evaluated using the AMSTAR 2 (A Measurement Tool to Assess Systematic Reviews, Version 2) tool. This validated tool assesses 16 domains, with an emphasis on seven critical areas, including protocol registration, adequacy of the literature search, justification for excluded studies, assessment of the risk of bias in included studies, and appropriateness of meta-analytical methods. Each review was assigned a quality rating based on the AMSTAR 2 guidelines. Studies with no or only one non-critical weakness were rated as high quality. Those with no critical flaws but some non-critical weaknesses were classified as moderate quality. Reviews with at least one critical flaw were rated as low quality, while those with multiple critical flaws were considered critically low quality [13].

The AMSTAR 2 assessments were conducted independently by two reviewers, with any discrepancies resolved through discussion or by consulting a third reviewer. This process provided a comprehensive evaluation of the reliability and validity of the included studies, ensuring methodological rigor.

### 2.6. Statistical Analysis

For data synthesis, we first conducted a qualitative (narrative) summary of the included systematic reviews to describe their main characteristics, scope, and findings, followed by quantitative meta-analytical pooling where applicable. The results of the included studies were pooled using random-effects models to account for variability in study design, sample characteristics, and outcome measures. The overall effect sizes were expressed as risk ratios (RRs) with 95% confidence intervals (CIs). The heterogeneity between the studies was assessed using the *I*^2^ statistic, with values greater than 50% indicating substantial heterogeneity.

Forest plots were generated using R software (version 4.4.2), employing the meta and metafor packages to visualize the pooled estimates and the heterogeneity [14]. These plots provided a graphical representation of the effect sizes for each study and the overall pooled effect size with its confidence intervals. Statistical significance was determined at a *p*-value of <0.05. All analyses adhered to the PRISMA guidelines to ensure methodological rigor and transparency.

## 3. Results

### 3.1. Selected Studies

**Figure 1:**
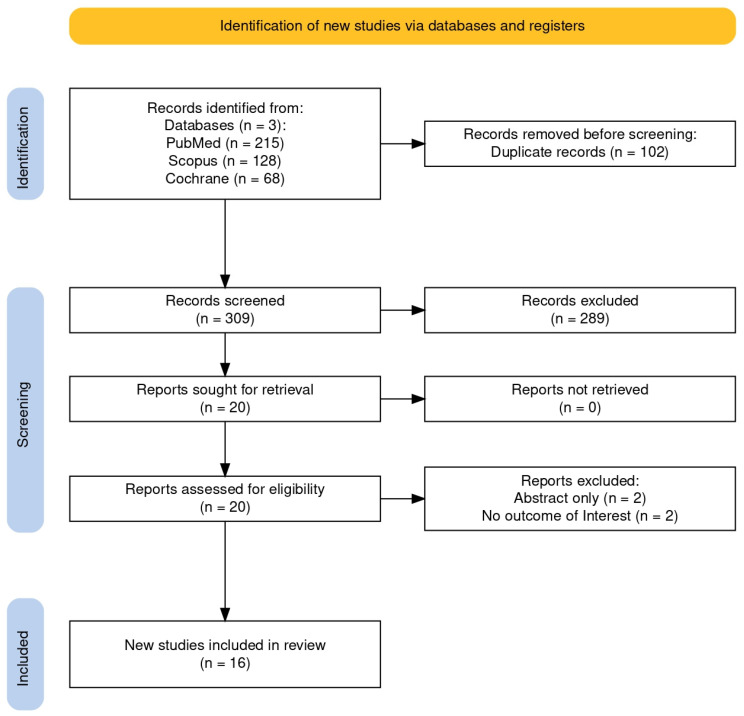
Search outputs based on PRISMA guidelines [16].

The systematic search across three databases, PubMed (*n* = 215), Scopus (*n* = 128), and the Cochrane Library (*n* = 68), yielded a total of 411 potentially eligible articles. After removing 102 duplicate records, 309 unique articles underwent abstract screening using the Rayyan platform. Of these, 289 articles were excluded due to irrelevance or non-compliance with the inclusion criteria during the title and abstract review. Subsequently, 20 articles underwent full-text assessment. Among these, four were excluded for the following reasons: two were conference abstracts without sufficient data, and two did not report the outcomes of interest. Ultimately, 16 studies met the inclusion criteria and were included in the umbrella review. The study selection process is depicted in Figure 1, generated using the web-based application developed by Haddaway et al. [15].

### 3.2. Study Characteristics

The characteristics of the 16 studies included in this umbrella review are summarized in Table 1. All studies were systematic reviews and/or meta-analyses, with publication years ranging from 2007 to 2024. The studies collectively included a total of 41,072 patients across diverse geographical locations, including Australia, China, Japan, and Canada. The included studies predominantly examined the effects of IMN or enteral immune-enhancing nutrition (EIN) on the patients undergoing elective GI cancer surgeries.

The interventions included preoperative, perioperative, and postoperative administration of IMN formulas. These formulas often contained components such as arginine, omega-3 fatty acids, glutamine, nucleotides, and RNA. The types of cancers studied encompassed gastric, colorectal, esophageal, pancreatic, and head and neck cancers. The majority of the studies were randomized controlled trials (RCTs), with sample sizes ranging from 606 to 6370 patients. This comprehensive synthesis highlights significant variability in study designs, populations, and intervention protocols, underscoring the need for careful interpretation of the findings across the studies.

### 3.3. Postoperative Infectious Complications

**Figure 2:**
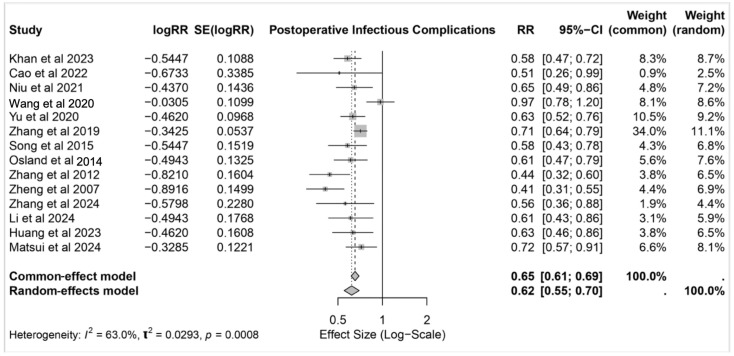
Forest plot for postoperative infectious complications [17,18,19,20,21,22,23,24,25,26,27,28,30,31].

The meta-analysis evaluated the effect of IMN on postoperative infectious complications across 16 studies. The pooled results from the random-effects model indicated that IMN significantly reduced the risk of postoperative infections, with a relative risk (RR) of 0.62 (95% CI: 0.55–0.70). These findings are consistent with the common-effect model estimate (RR: 0.65, 95% CI: 0.61–0.69) (Figure 2).

Notably, the heterogeneity among the included studies was moderate (*I*^2^ = 63.0%, *p* = 0.0008), suggesting some variation in the study outcomes. Despite this, the direction of the effect was uniformly in favor of IMN across all studies, with relative risks consistently below 1.

The individual studies showed varying weights, with Zhang et al. [22] contributing the greatest weight (11.1% in the random-effects model), while smaller weights were observed for studies like Cao et al. [18] and Matsui et al. [31]. Across the spectrum of studies, the intervention consistently demonstrated a protective effect against postoperative infectious complications in the patients undergoing gastrointestinal cancer surgeries.

#### 3.3.1. Pneumonia

**Figure 3:**
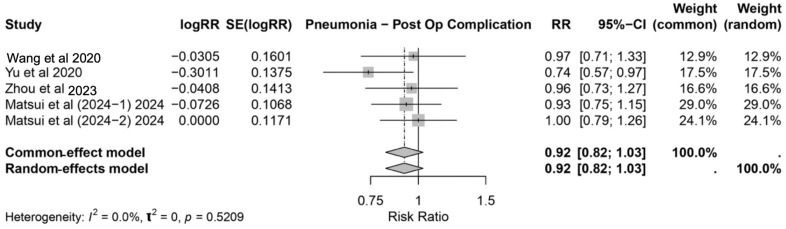
Forest plot showing the pooled relative risk (RR) and 95% confidence intervals for postoperative pneumonia with immunonutrition across five studies [3,20,21,29,31].

The association between IMN and the risk of postoperative pneumonia was evaluated across five studies [3,20,21,29,31]. The pooled relative risk (RR) from both the common-effect and random-effects models was 0.92 (95% CI: 0.82–1.03), indicating no statistically significant reduction in pneumonia risk with IMN (Figure 3). The individual study estimates displayed varying effect sizes, with Yu et al. [21] showing the most notable reduction in risk (RR: 0.74, 95% CI: 0.57–0.97). However, other studies, such as Matsui et al. [31], reported no significant effect (RR: 1.00, 95% CI: 0.79–1.26). The heterogeneity among the included studies was negligible, as indicated by *I*^2^ = 0.0% (*p* = 0.5209), confirming consistency in the study outcomes.

#### 3.3.2. Urinary Tract Infection

**Figure 4:**
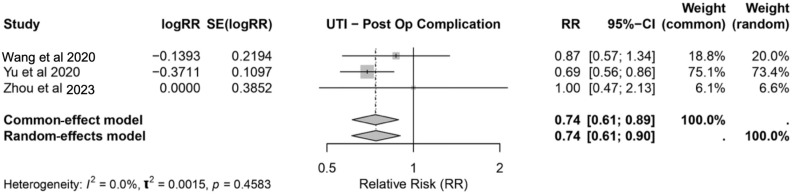
Forest plot showing the pooled RR and 95% confidence intervals for postoperative urinary tract infections with immunonutrition [20,21,29].

The impact of IMN on postoperative urinary tract infections was assessed across three studies [20,21,29]. The pooled analysis revealed a significant reduction in the risk of UTIs with IMN, with an RR of 0.74 (95% CI: 0.61–0.89) based on both the common-effect and random-effects models. The heterogeneity among the studies was minimal (*I*^2^ = 0.0%, *p* = 0.4583), indicating consistency in the reported outcomes. These findings suggest that IMN may provide a protective effect against this specific postoperative complication (Figure 4). Overall, the results demonstrate a consistent reduction in postoperative UTI risk among the patients receiving IMN, further supporting its beneficial role in mitigating infectious complications after surgery.

#### 3.3.3. Wound Infection

The meta-analysis assessed the impact of IMN on wound infection rates across seven studies [17,19,20,21,26,27,29]. The pooled results demonstrated a significant reduction in wound infections among the patients receiving IMN, with an OR of 0.64 (95% CI: 0.55–0.73) under the random-effects model. These findings were consistent with the common-effect model estimate (OR: 0.65, 95% CI: 0.58–0.72). The heterogeneity was low to moderate (*I*^2^ = 34.4%, *p* = 0.1654), reflecting some variability across the studies. Among the included studies, Yu et al. [21] contributed the highest weight (26.1% in the random-effects model), while smaller weights were observed for Wang et al. [20] and Zhou et al. [29].

**Figure 5:**
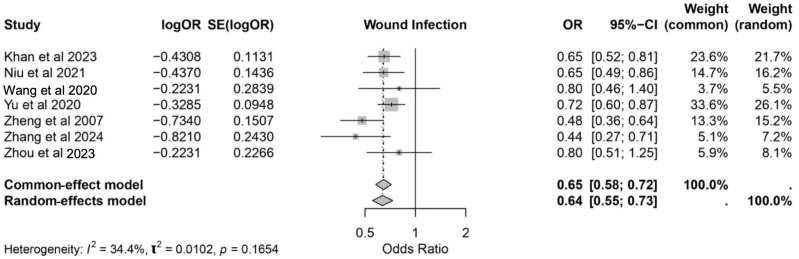
Forest plot showing the effect of immunonutrition on wound infection rates in postoperative patients [17,19,20,21,26,27,29].

The patients who had received IMN exhibited enhanced wound healing, as evidenced by the reduced wound infection rates and the improved recovery outcomes. Nutritional formulations enriched with arginine, glutamine, and omega-3 fatty acids were particularly associated with these benefits. These findings highlight the role of IMN in optimizing postoperative wound recovery, especially in the patients undergoing major gastrointestinal surgeries (Figure 5).

### 3.4. Postoperative Noninfectious Complications

**Figure 6:**
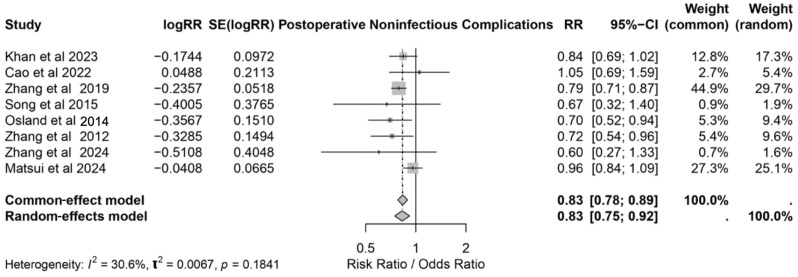
Forest plot showing the effect of immunonutrition on postoperative noninfectious complications with pooled risk ratios under the common- and random-effects models [17,18,22,23,24,25,27,31].

The meta-analysis evaluated the effect of IMN on postoperative non-infectious complications across eight studies [17,18,22,23,24,25,27,31]. The pooled results showed a significant reduction in non-infectious complications among the patients receiving IMN, with an RR of 0.83 (95% CI: 0.75–0.92) under the random-effects model. These findings were consistent with the common-effect model estimate (RR: 0.83, 95% CI: 0.78–0.89). The heterogeneity was low (*I*^2^ = 30.6%, *p* = 0.1841), indicating limited variability across the studies (Figure 6). IMN was associated with a reduced risk of anastomotic leaks, wound dehiscence, and respiratory complications, highlighting its role in minimizing postoperative non-infectious complications. These benefits underscore the potential of IMN for improving surgical outcomes, particularly in high-risk patients undergoing major procedures.

### 3.5. Anastomotic Leaks

**Figure 7:**
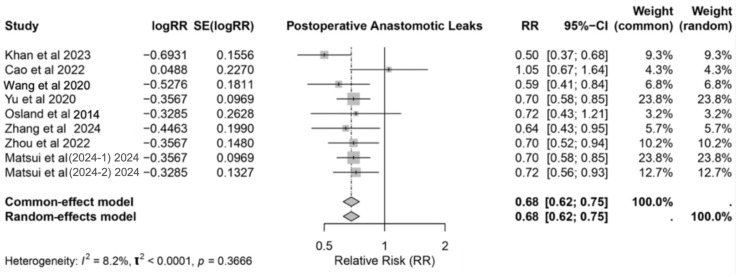
Forest plot showing postoperative anastomotic leaks with immunonutrition [3,17,18,20,21,24,27,29,31].

The pooled analysis of nine studies [3,17,18,20,21,24,27,29,31] demonstrated that IMN significantly reduced the risk of postoperative anastomotic leaks, with an RR of 0.68 (95% CI: 0.62–0.75) under both the common-effect and the random-effects models. The forest plot (Figure 7) illustrates the consistency of the findings across the studies, with low heterogeneity (*I*^2^ = 8.2%, *p* = 0.3666). Khan et al. [17] reported the most substantial reduction in risk (RR: 0.50, 95% CI: 0.37–0.68), while Cao et al. [18] showed a non-significant effect (RR: 1.05, 95% CI: 0.67–1.64). Studies with higher weights, such as Yu et al. [21] and Matsui et al. [31], provided more robust evidence supporting the protective effect of IMN, with RRs of 0.70 (95% CI: 0.58–0.85) and 0.72 (95% CI: 0.56–0.93), respectively.

These findings highlight the role of IMN in mitigating anastomotic leaks, a critical complication associated with increased morbidity and extended hospital stays. The reduction in risk underscores the potential of IMN as a perioperative intervention to improve surgical outcomes in gastrointestinal cancer patients.

### 3.6. Length of Hospital Stay

**Figure 8:**
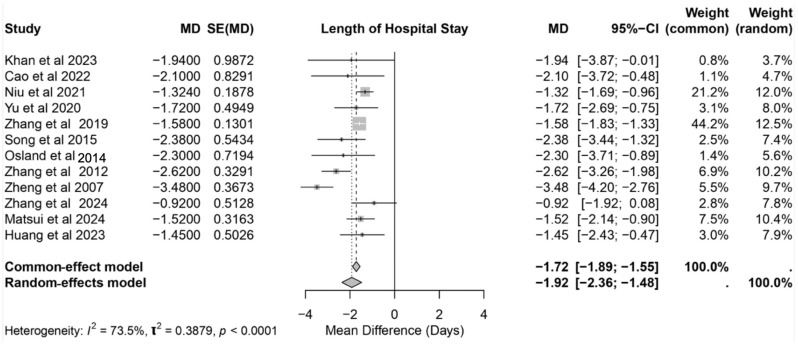
Forest plot showing the length of hospital stay (in days) with immunonutrition across 12 studies [17,18,19,21,22,23,24,25,26,27,30,31].

The effect of IMN on the length of the hospital stay was evaluated across 12 studies, as shown in Figure 8. The pooled mean difference (MD) under the random-effects model was –1.92 days (95% CI: −2.36 to −1.48; *p* < 0.0001), indicating a statistically significant reduction in the length of the hospital stay for the patients receiving IMN compared to the controls. The heterogeneity among the studies was high (*I*^2^ = 73.5%, T^2^ = 0.3879), suggesting substantial variability in the effect sizes. The studies by Zheng et al. [26] and Song et al. [23] reported the greatest reductions in hospital stay, with MDs of –3.48 days (95% CI: −4.20 to −2.76) and −2.38 days (95% CI: −3.44 to −1.32), respectively. By contrast, Zhang et al. [27] reported the smallest effect (MD: −0.92 days, 95% CI: −1.92 to 0.08). Despite the variability, the consistent trend across most studies supports the beneficial impact of IMN in reducing hospitalization duration.

### 3.7. Mortality Rate

**Figure 9:**
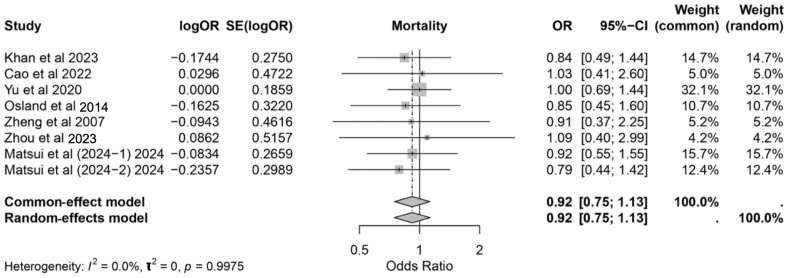
Forest plot showing the effect of immunonutrition on mortality with the pooled odds ratio (OR) and 95% confidence intervals (CIs) [3,17,18,21,24,26,29,31].

The impact of IMN on mortality was assessed in eight studies, as illustrated in Figure 9. The pooled OR using the random-effects model was 0.92 (95% CI: 0.75–1.13; *p* = 0.9975), indicating no statistically significant difference in the mortality between the IMN and the control groups. The heterogeneity among the studies was negligible, reflecting consistency in the effect sizes across the included studies. Most individual studies, such as Khan et al. [17] and Osland et al. [24], reported ORs close to 1, with confidence intervals crossing the null, indicating no significant effect. Similarly, the studies by Yu et al. [21] and Matsui et al. [3] showed neutral outcomes. The lack of significant association suggests that IMN does not influence the mortality rates in the studied populations.

### 3.8. Nutritional Status Improvement

Among the sixteen studies included in this review, seven evaluated the impact of perioperative IMN on postoperative nutritional status, with five providing robust statistical evidence of improvement. These studies highlighted the superior ability of IMN, compared to conventional nutritional support, to modulate inflammatory responses, to reduce pro-inflammatory cytokines, and to enhance immune cell counts, thereby strengthening overall immune system functionality.

For inflammatory markers, Niu et al. [19] reported a significant reduction in the *C*-reactive protein (CRP) levels, with a Hedges’ g of −1.529 (95% CI [−2.250, −0.809], *p* < 0.001). Conversely, Zheng et al. [26] did not observe significant changes in the CRP levels (WMD = −12.70, 95% CI [−32.17–2.77], *p* = 0.20).

Regarding lymphocyte counts, Zheng et al. [26] and Huang et al. [30] both documented significant increases. Zheng et al. [26] reported a WMD of 0.40 (95% CI [0.21–0.59], *p* < 0.00001), while Huang et al. observed an SMD of 1.34 (95% CI [0.39–3.07], *p* = 0.0001). Notably, Zheng et al. [26] also demonstrated significant elevations in the CD4 cell counts (WMD = 11.39, 95% CI [6.20–16.58], *p* < 0.00001). However, Li et al. [28] found no significant changes in the CD4 levels (MD = 2.92, 95% CI [−3.84 to 9.69], I^2^ = 99%), but they reported a significant increase in the CD8 cell counts (MD = 1.16, 95% CI [0.06–2.26], *p* = 0.04, *I*^2^ = 0%) and an improved CD4/CD8 ratio (MD = 0.48, 95% CI [0.08–0.88], *p* = 0.02, *I*^2^ = 92%).

Immunoglobulin levels also improved with IMN. Li et al. [28] observed a significant increase in the IgG levels (MD = 2.46, 95% CI [1.47–3.46], *p* < 0.001). Similarly, Zhang et al. [27] reported significant increases in the IgA (MD = 0.58, 95% CI [0.31–0.85], *p* < 0.01, *I*^2^ = 58%), the IgG (MD = 1.67, 95% CI [1.15–2.19], *p* < 0.01, *I*^2^ = 0%), and the IgM levels (MD = 0.40, 95% CI [0.20–0.61], *p* < 0.01, *I*^2^ = 77%). Zhang et al. [27] also noted the enhancements in tumor-infiltrating lymphocyte counts, with significant increases in the CD16 (MD = 0.04, 95% CI [0.02–0.06], *p* < 0.001) and the CD56 levels (MD = 0.05, 95% CI [0.03–0.06], *p* < 0.001).

These findings collectively highlight the role of IMN in improving postoperative nutritional and immune status by effectively regulating inflammatory responses and enhancing immune function. Table 2 provides a detailed summary of these outcomes.

### 3.9. Quality Assessment

A total of 16 manuscripts were included in this umbrella review and were assessed using the AMSTAR 2 online tool [13]. Five manuscripts received a low score [18,21,22,27,30], while eleven were rated as critically low [3,17,19,20,23,24,25,26,28,29,31]. The common shortcomings included the absence of a pre-specified protocol [19,20,23,24,25,26,29,30,31] and failure to justify the inclusion of only RCTs [19,21,25,26,29,30]. Although all studies partially addressed the comprehensive search strategy domain, most lacked key elements, such as searching trial registries or reference lists. Only one manuscript provided a partial list of the excluded articles with the reasons for exclusion, and just two reported the funding sources for the included trials [17,24] (Table 3).

While most studies adhered to the PICO criteria and performed duplicate study selection and data extraction, two did not [19,20]. All manuscripts disclosed the author conflicts of interest and/or funding. However, critical domains were frequently unmet, significantly affecting the quality scores. Inconsistencies in evaluating the risk of bias and the heterogeneity were common, with limited discussion of these findings in the manuscripts’ results or conclusions.

The variability in quality among the selected manuscripts represents a limitation of this review. Although AMSTAR 2 offers an objective framework, assessing manuscript quality remains somewhat subjective. Applying the tool during the initial screening could help establish quality as an inclusion criterion. Greater familiarity with AMSTAR 2′s components may guide authors in producing higher-quality manuscripts for inclusion in meta-analyses. Additionally, introducing more “partial yes” responses in the tool could enhance its granularity, providing a more nuanced assessment of quality and aiding interpretation.

## 4. Discussion

This umbrella review and meta-analysis provides a comprehensive synthesis of the evidence for the perioperative role of IMN in GI cancer surgery. By integrating data from 16 high-quality systematic reviews and meta-analyses, we demonstrate the clinical utility of IMN in reducing postoperative complications and enhancing recovery. The key findings include significant reductions in infectious complications, such as SSI and UTI, as well as non-infectious complications like anastomotic leaks, and wound dehiscence. Moreover, IMN was associated with improved wound healing, enhanced immune function, and better maintenance of nutritional parameters, such as lymphocyte counts and albumin levels. The observed reductions in hospital length of stay further highlight its potential to optimize healthcare resource utilization.

Postoperative infectious complications, including SSI, pneumonia, and UTI, remain as a major concern in surgical oncology due to their association with increased morbidity, prolonged hospitalization, and elevated healthcare costs [32]. This review underscores the significant role of IMN in mitigating these risks, with a pooled relative risk (RR) of postoperative complications of 0.62 (95% CI: 0.55–0.70). Specifically, IMN was associated with a 36% reduction in SSI risk (OR: 0.64, 95% CI: 0.55–0.73) and a 26% reduction in UTI risk (RR: 0.74, 95% CI: 0.61–0.89), supporting its immunomodulatory effects. These effects likely stem from the synergistic action of nutrients such as arginine, omega-3 fatty acids, and glutamine [33].

The weight of individual studies varied, with Zhang et al. [22] contributing the largest weight in the random-effects model (11.1%), while studies like Cao et al. [18] and Matsui et al. [31] had smaller contributions. Despite this variability, the data robustly support the role of IMN in enhancing immune function, regulating inflammatory responses, and promoting tissue repair, leading to improved surgical outcomes.

Interestingly, IMN did not significantly reduce the risk of pneumonia (RR: 0.92, 95% CI: 0.82–1.03). Although Yu et al. [21] reported a notable protective effect (RR: 0.74, 95% CI: 0.57–0.97), other studies, such as Matsui et al. [31], demonstrated no significant benefit (RR: 1.00, 95% CI: 0.79–1.26). This highlights the multifactorial nature of pneumonia prevention, which likely depends on comprehensive perioperative management strategies, including pulmonary care protocols, beyond the scope of IMN alone.

The role of IMN in reducing non-infectious complications, particularly anastomotic leaks (RR: 0.68, 95% CI: 0.62–0.75), is another critical finding. Anastomotic leaks are severe complications with significant implications for patient morbidity and mortality. Khan et al. [17] reported the most substantial reduction in risk (RR: 0.50, 95% CI: 0.37–0.68), whereas Cao et al. [18] found no significant effect (RR: 1.05, 95% CI: 0.67–1.64). Larger, higher-weight studies, such as Yu et al. [21] and Matsui et al. [31], corroborated the protective effects, with RRs of 0.70 (95% CI: 0.58–0.85) and 0.72 (95% CI: 0.56–0.93), respectively. These findings underscore IMN’s potential to preserve intestinal integrity, to promote tissue repair, and to enhance surgical outcomes, particularly for high-risk patients [34].

The significant reduction in hospital length of stay (MD: −1.92 days, 95% CI: −2.36 to −1.48) further underscores the clinical benefits of IMN. Variability was noted among the studies, with the greatest reductions reported by Zheng et al. [26] (MD: −3.48 days, 95% CI: −4.20 to −2.76) and Song et al. [23] (MD: −2.38 days, 95% CI: −3.44 to −1.32), while Zhang et al. [27] observed the smallest effect (MD: −0.92 days, 95% CI: −1.92 to 0.08). These outcomes likely reflect IMN’s role in reducing complications, enhancing wound healing, and facilitating earlier discharge.

Maintaining optimal nutritional and immune status is pivotal for surgical recovery. This review highlights IMN’s efficacy in improving the markers of nutritional and immune status, such as reductions in the *C*-reactive protein levels and enhancements in the lymphocyte counts and the immunoglobulin levels. These findings reinforce the importance of personalized nutritional interventions to meet perioperative metabolic demands and to optimize patient outcomes.

Despite the promising findings, several limitations must be acknowledged. The included studies exhibited heterogeneity in the intervention protocols, patient populations, and study designs. Variability in IMN formulations, dosages, and administration durations likely contributed to inconsistencies in the outcomes. Moreover, the inclusion of studies spanning a broad timeframe (2007 to 2024) may reflect the temporal changes in surgical practices, perioperative care, and the composition of IMN formulas, potentially increasing clinical and methodological heterogeneity. While this extended period enhances the comprehensiveness of the synthesis, it also introduces variation that must be interpreted with caution. Additionally, due to variability in the outcomes assessed across the included meta-analyses, certain complication-specific analyses (e.g., pneumonia, UTI) were based on a smaller number of studies. This limitation may affect the generalizability and statistical power of these specific findings, and highlights the need for more standardized outcome reporting in future research. Furthermore, critical methodological shortcomings, as identified through AMSTAR 2, include the lack of pre-specified protocols and the incomplete reporting of funding sources. Addressing these issues in future research will be essential to enhance the robustness and applicability of the evidence. Future research should prioritize large-scale, rigorously designed randomized controlled trials to standardize the IMN protocols, to refine the patient selection criteria, and to evaluate the long-term outcomes. Investigating the role of IMN within neoadjuvant and adjuvant therapy regimens could offer additional insights into its integration into comprehensive cancer care. A focus on cost-effectiveness analyses and quality-of-life metrics will further inform clinical and policy decisions.

## 5. Conclusions

This umbrella review highlights the significant clinical benefits of perioperative IMN in gastrointestinal cancer surgery, including reductions in infectious and non-infectious complications, improved nutritional and immune status, and shorter hospital stays. Despite its promise, variability in the study protocols and the patient populations underscore the need for cautious interpretation and targeted application. Future research should focus on standardizing the IMN protocols, refining the patient selection criteria, and conducting large-scale, high-quality randomized controlled trials to further validate its role in optimizing the surgical outcomes and enhancing perioperative care.

## Figures and Tables

**Table 1:** Study characteristics of the included studies.

Authors	Year of Publication	Study Design	Country	Number of Studies Included	Time Period	Total Number of Patients Included	Type of Surgery	Type of Cancer	Interventions
Khan et al. [17]	2023	SR & MA	Australia	37 RCT	2000–2022	3793	Elective GI Cancer	Upper GI, colorectal, and mixed GI cancers	Preoperative, perioperative, and postoperative enteral IMN: Arg, glutamine, ω-3-FA, and nucleotides.
Cao et al. [18]	2022	SR & MA	China	5 RCTs and 10 observational	1991–2018	1864	Esophagectomy	Esophageal cancer	Preoperative IEN: Arg, ω-3-FA, and nucleotides
Niu et al. [19]	2021	SR & MA	China	25 RCT	1988–2011	2238	Elective GI Cancer	Gastric, colorectal, and pancreatic cancers	Perioperative IMN: Arg, ω-3-FA, and nucleotides
Wang et al. [20]	2020	SR	China	11 RCT	2007–2019	606	Esophagectomy	Esophageal Cancer	Perioperative EIN: Arg, RNA, and ω-3-FA (formulas like IMPACT, EPA, Oxepa)
Yu et al. [21]	2020	SR & MA	China	61 RCT	1994–2017	5983	Elective surgical resection for gastrointestinal cancers.	Gastric, Colorectal, Esophageal, and Pancreatic	Perioperative IMN, including Arg, ω-3-FA, and RNA, compared to standard nutrition.
Zhang et al. [22]	2019	SR & MA	Canada	56 RCT	1995–2018	6370	Elective GI Cancer	Gastric, colorectal, esophageal, and pancreatic cancer	Perioperative IMN: Arg, ω-3-FA, RNA, protein supplementation, and carbohydrate
Song et al. [23]	2015	SR & MA	China	27 RCT	1992–2013	2538	Elective GI Cancer	Gastric, colorectal, esophageal, and pancreatic cancers	Preoperative, perioperative, and postoperative IMN: Arg, ω-3-FA, glutamine, and RNA
Osland et al. [24]	2013	SR & MA	Australia	20 RCT	1988–2011	2005	Elective GI Cancer	Gastric, pancreatic, esophageal cancers	Arg-dominant pharmaconutrition vs standard enteral nutrition, delivered preoperatively, perioperatively, or postoperatively.
Zhang et al. [25]	2012	SR & MA	China	19 RCT	1995–2011	2331	Elective GI Cancer	Gastric, colorectal, pancreatic, and esophageal cancers	Perioperative IMN: Arg, ω-3-FA, glutamine, RNA
Zheng et al. [26]	2007	MA	China	13 RCT	1992–2005	1269	Elective GI Cancer	Gastric, colorectal, and pancreatic cancers	Perioperative IMN: Arg, glutamine, ω-3-FA, and RNA
Zhang et al. [27]	2024	SR & MA	China	16 RCTs	2002–2023	1416 patients	Colorectal cancer surgery	Colorectal cancer	Preoperative IMN: Arg, ω-3-FA, glutamine, RNA
Li et al. [28]	2024	SR & MA	China	12 RCT	2005–2022	1124	Gastrectomy	Gastric Cancer	IMN: ω-3-FA, Arg, glutamine, nucleotides
Zhou et al. [29]	2022	SR & MA	China	10 RCT	2007–2020	1052	Esophagectomy	Esophageal Cancer	EIN: Arg, glutamine, ω-3-FA and nucleotides
Matsui et al. [3]	2024	SR & MA	Japan	48 RCT	1981–2022	4825	Elective Heck and Neck Cancer Surgery and Elective GI Cancer	Head and Neck Gastrointestinal Cancer	Preoperative, perioperative, and postoperative enteral IMN: Arg, ω-3-FA, or glutamine
Huang et al. [30]	2023	MA	China	10 RCT	2005–2022	1409	Gastrectomy	Gastric Cancer	EIN: ω-3 fatty acids, glutamine, Arg, and nucleotides.
Matsui et al. [31]	2024	SR & MA	Japan	23 RCT	2000–2022	2249	Elective GI Cancer	Upper GI cancer	Preoperative, perioperative, and postoperative enteral IMN: Arg, ω-3-FA, or glutamine

SR: Systematic Review; MA: Meta-Analysis; ω-3-FA: omega-3 fatty acid; Arg: arginine; IMN: immunonutrition; IEN: immune-enhancing nutrition; EIN: enteral immunonutrition.

**Table 2:** Key Findings on Nutritional and Immune Status Improvement.

Study	Parameter	Effect Size	95% CI	*p*-Value
Niu et al., 2021 [19]	CRP	Hedges’ g = −1.529	[−2.250–0.809]	<0.001
Zheng et al., 2007 [26]	Lymphocytes	WMD = 0.40	[0.21–0.59]	<0.00001
	CD4	WMD = 11.39	[6.20–16.58]	<0.00001
Huang et al., 2023 [30]	Lymphocytes	SMD = 1.34	[0.39–3.07]	0.0001
Li et al., 2024 [28]	CD4	MD = 2.92	[−3.84 to 9.69]	Not significant
	CD8	MD = 1.16	[0.06–2.26]	0.04
	CD4/CD8 ratio	MD = 0.48	[0.08–0.88]	0.02
	IgG	MD = 2.46	[1.47–3.46]	<0.001
Zhang et al., 2024 [27]	IgA	MD = 0.58	[0.31–0.85]	<0.01
	IgG	MD = 1.67	[1.15–2.19]	<0.01
	IgM	MD = 0.40	[0.20–0.61]	<0.01
	CD16	MD = 0.04	[0.02–0.06]	<0.001
	CD56	MD = 0.05	[0.03–0.06]	<0.001

**Table 3:** AMSTAR 2 evaluation for each domain for every manuscript included in the Umbrella Review.

AMSTAR 2 Domain	Khan et al. [17]	Cao et al. [18]	Niu et al. [19]	Wang et al. [20]	Yu et al. [21]	Zhang et al. [22]	Song et al. [23]	Osland et al. [24]	Zhang et al. [25]	Zheng et al. [26]	Zhang et al. [27]	Li et al. [28]	Zhou et al. [29]	Matsui et al. [3]	Huang et al. [30]	Matsui et al. [31]
**1**	Y	Y	N	N	Y	Y	Y	Y	Y	Y	Y	Y	Y	Y	Y	Y
**2**	Y	Y	N	N	Y	Y	N	N	N	N	Y	Y	N	Y	PY	PY
**3**	Y	Y	N	Y	N	Y	Y	Y	N	N	Y	Y	N	Y	N	Y
**4**	PY	PY	PY	PY	PY	PY	PY	PY	PY	PY	PY	PY	PY	PY	PY	PY
**5**	Y	Y	Y	Y	Y	Y	Y	Y	Y	Y	Y	Y	Y	Y	Y	Y
**6**	Y	Y	Y	Y	Y	Y	Y	Y	Y	Y	Y	Y	Y	Y	Y	Y
**7**	N	N	N	N	N	N	N	N	N	N	PY	N	N	N	N	N
**8**	Y	Y	Y	Y	Y	PY	Y	Y	Y	Y	Y	Y	Y	N	Y	Y
**9**	Y	Y	N	Y	Y	Y	Y	N	Y	N	Y	PY	Y	PY	PY	Y
**10**	Y	N	N	N	N	N	N	Y	N	N	N	N	N	N	N	N
**11**	Y	Y	N	Y	Y	Y	Y	Y	Y	N	Y	Y	Y	Y	Y	Y
**12**	Y	Y	N	N	Y	Y	Y	N	N	N	Y	N	Y	Y	N	N
**13**	Y	Y	N	Y	Y	Y	Y	N	N	N	N	N	N	N	Y	N
**14**	Y	Y	N	Y	Y	Y	Y	Y	N	N	Y	N	N	N	N	N
**15**	N	Y	Y	Y	Y	Y	N	Y	N	N	Y	Y	N	Y	Y	Y
**16**	Y	Y	Y	Y	Y	Y	Y	Y	Y	Y	Y	Y	Y	Y	Y	Y
**Score**	Critical	Low	Critical	Critical	Low	Low	Critical	Critical	Critical	Critical	Low	Critical	Critical	Critical	Low	Critical

Y: Yes; N: No; PY: Partial Yes.

## Data Availability

The datasets generated and/or analyzed during this study are not publicly available but may be obtained from the corresponding author upon reasonable request.
